# Impact of a new cardiac magnetic resonance (CMR) program on management and clinical decision-making in cardiomyopathy patients

**DOI:** 10.1186/1532-429X-14-S1-P144

**Published:** 2012-02-01

**Authors:** Siddique Abbasi, Jaehoon Chung, Geetha Bhat, Ankit A Desai, Thomas Stamos, Melissa Robinson Wood, Joan Briller, George T Kondos, Samuel Dudley, Afshin Farzaneh-Far

**Affiliations:** 1 Division of Cardiology, University of Illinois at Chicago, Chicago, IL, USA; 2 Center for Heart Transplant and Assist Devices, Advocate Christ Medical Center, Oak Lawn, IL, USA; 3 Division of Cardiology, Duke University, Durham, NC, USA

## Summary

We sought to evaluate the direct impact of a new CMR program on clinical management and decision-making in patients with cardiomyopathies from our established Heart Failure program.

## Background

CMR provides unique diagnostic and prognostic information in patients with cardiomyopathies. Despite this, widespread uptake of CMR by Heart Failure physicians has been slow, particularly in the United States. In the current health care climate, routine use of a new imaging modality like CMR requires evidence for direct additive impact on clinical management.

## Methods

This was a single-center registry from an academic medical center in the United States. The first 200 patients from a newly established CMR program were enrolled. All procedures were performed using standardized SCMR-recommended protocols on 1.5T and 3T scanners and interpreted by a Level 3 reader. Definitions for “significant clinical impact” of CMR were pre-defined and were collected directly from medical records and/or from patients. Categories of clinical impact included: new diagnosis, medication change, hospital admission/discharge, as well as performance or avoidance of invasive procedures (angiography, revascularization, device therapy or biopsy).

## Results

No complications occurred and image quality was good in 96% of cases. Overall, 36% (n=75) of referrals were for assessment of cardiomyopathy (ejection fraction <45%). All cardiomyopathy patients had a recent prior echocardiogram. In approximately two-thirds (63%) of these patients, CMR had a significant impact on clinical management and decision-making. This included an entirely new diagnosis in 23% of cases and a change in medication management in 15%. Anticoagulation was stopped in 5% and started in 3%. CMR results directly led to angiography in 7% and to the performance of percutaneous coronary intervention in 7%. Bypass-surgery was performed in 4% and avoided in 9% directly as a result of CMR findings. Likewise, defibrillator placement was performed in 5% and avoided in 4%.

## Conclusions

CMR makes a significant impact on clinical management and decision-making in cardiomyopathy patients from an established Heart Failure program. It led to a significant change in clinical management in 63% of patients referred for assessment of cardiomyopathy. This additive impact was seen despite widespread use of echocardiography and other imaging tests in this patient group. This study lends support to the growing use of CMR by Heart Failure programs in the United States.

## Funding

None.

